# The Diagnostic Value of Transcription Factors T-bet/GATA3 Ratio in Predicting Antibody-Mediated Rejection

**DOI:** 10.1155/2013/460316

**Published:** 2013-10-23

**Authors:** Xue Li, Qiquan Sun, Mingchao Zhang, Jinsong Chen, Zhihong Liu

**Affiliations:** ^1^Research Institute of Nephrology, Jinling Hospital, Nanjing University Clinical School of Medicine, 305 East Zhongshan Road, Nanjing 210002, China; ^2^Department of Renal Transplantation, The Third Affiliated Hospital, Sun Yat-sen University, 2693 Kaichuang Avenue, Luogang District, Guangzhou 510530, China

## Abstract

*Background*. Previous data showed that the predominance of intraglomerular T-bet or GATA3 is correlated with different mechanisms of rejection, suggesting that the ratio of T-bet/GATA3 might be used to distinguish antibody-mediated rejection (ABMR) and T-cell-mediated rejection (TCMR). *Methods*. We compared the intraglomerular T-bet/GATA3 ratio in ABMR and TCMR. The intragraft expression of T-bet and GATA3 was studied via immunohistochemistry. The correlation of the diagnosis of AMR with the ratio of T-bet/GATA3 was examined. *Results*. Both intraglomerular T-bet- and GATA3-expressing cells were increased during acute rejection. T-bet/GATA3>1 was strongly correlated with ABMR (93.3% versus 18.2%). The incidence of positive HLA-I/II antibodies and glomerulitis is significantly higher in T-bet/GATA3>1 group (*P* < 0.001, 0.013, resp.). The scores of peritubular capillary inflammation and glomerulitis were also higher in T-bet/GATA3>1 group (*P* = 0.052, *P* < 0.001, resp.). Nevertheless, T-bet/GATA3>1 is also correlated with C4d-negative ABMR and resistance to steroid treatment. Compared with C4d deposition, T-bet/GATA3>1 had a slight lower (90% versus 100%) specificity but a much higher (87.5% versus 68.8%) sensitivity. *Conclusion*. Our data suggested that intraglomerular predominance of T-bet over GATA3 might be used as diagnosis maker of ABMR in addition to C4d, especially in C4d-negative cases.

## 1. Introduction

With the introduction of new strong immunosuppressants, the incidence of early acute rejection is decreasing; however, antibody-mediated rejection (ABMR) remains an important barrier to successful renal transplantation [1–5]. Peritubular capillaries (PTC) C4d deposition was found to have high sensitivity and specificity for circulating antidonor antibodies and is regarded as a marker for ABMR in renal allograft recipients [4, 6]. It has been proposed that an early posttransplant biopsy displaying diffuse C4d-positive staining is suggestive of ABMR, even in the absence of detectable serum antihuman leukocyte antigen (HLA) antibodies [7].

However, accumulating evidence suggests that C4d deposition occurs only in some but not all ABMRs [8, 9]. A number of C4d-negative ABMR had been recognized. Conversely, C4d can also be detected in grafts with stable function [10]. Therefore, C4d is no longer a reliable maker of ABMR. Seeking a new marker for the diagnosis of ABMR is becoming more and more important. T-box expressed in T cells (T-bet) and GATA3 are two transcription factors that determine the T-helper cell differentiation into Th1 or Th2, respectively [11, 12]. The expression of T-bet had been reported to be increased in renal allograft developing acute rejection, and predominance of intraglomerular T-bet has also been observed in patients with antibody-mediated chronic rejection and transplant glomerulopathy [13, 14]. Our previous study [15] examined the expression of T-bet/GATA3 within the renal allografts. We found a predominant intraglomerular expression of T-bet in ABMR patients that was distinct from that in TCMR patients. In ABMR, there is a predominant expression of intraglomerular T-bet over GATA3, while GATA3 expression is predominant in TCMR. The predominance of intraglomerular T-bet expression relative to GATA3 expression was associated with poor response to bolus steroid treatment. These data suggest that the ratio of T-bet/GATA3 might be used to distinguish between ABMR and TCMR. This study was performed to evaluate the significance of intraglomerular T-bet/GATA3>1 as marker of ABMR, especially in the diagnosis of C4d-negative ABMR.

## 2. Concise Methods

### 2.1. Patients Selection

This study included twenty-six renal allograft recipients who were diagnosed as having acute rejection during 2006–2009. The diagnoses of ABMR and TCMR were based on Banff, 2001 [6]. Acute rejection which meets the diagnosis criteria of ABMR except for C4d deposition was diagnosed as C4d-negative ABMR; in addition, all C4d-negative ABMR episodes were required to occur within the first week after transplant and have severe graft dysfunction. All the rejection episodes were proven by renal biopsy. Informed consent was obtained from all patients, and the Human Subjects Committee of Jinling Hospital, Nanjing University School of Medicine, approved all study protocols.

### 2.2. Renal Biopsies

Renal biopsies were performed after onset of presumed rejection. Two needle biopsy cores were obtained from each renal allograft for morphologic study: one for formalin fixation and the other for quick freezing. Hematoxylin and eosin, periodic acid Schiff, methenamine-silver, and Masson stain were routinely used on the formalin-fixed tissue. The residual biopsy tissues were stored for future use. Fresh frozen tissue was analyzed by immunofluorescence microscopy using a conventional panel of antibodies against IgG, IgM, IgA, C3, C4, C1q, and C4d. C4d staining was routinely performed on frozen slides, using an indirect immunofluorescence technique with a primary affinity-purified monoclonal antibody (mouse antihuman; dilution, 1 : 50; 1.5-hour incubation at room temperature; Quidel, San Diego, CA) and an FITC-labeled affinity-purified secondary rabbit anti-mouse IgG antibody (1 : 20; 40 min incubation at room temperature; DAKO, Denmark). Staining was performed according to standard procedures. A positive C4d staining was defined as bright linear stain along capillary basement membranes, involving over half of sampled capillaries according to the 2001 Banff Meeting [6].

### 2.3. Immunohistological Analysis

**Figure 1:**
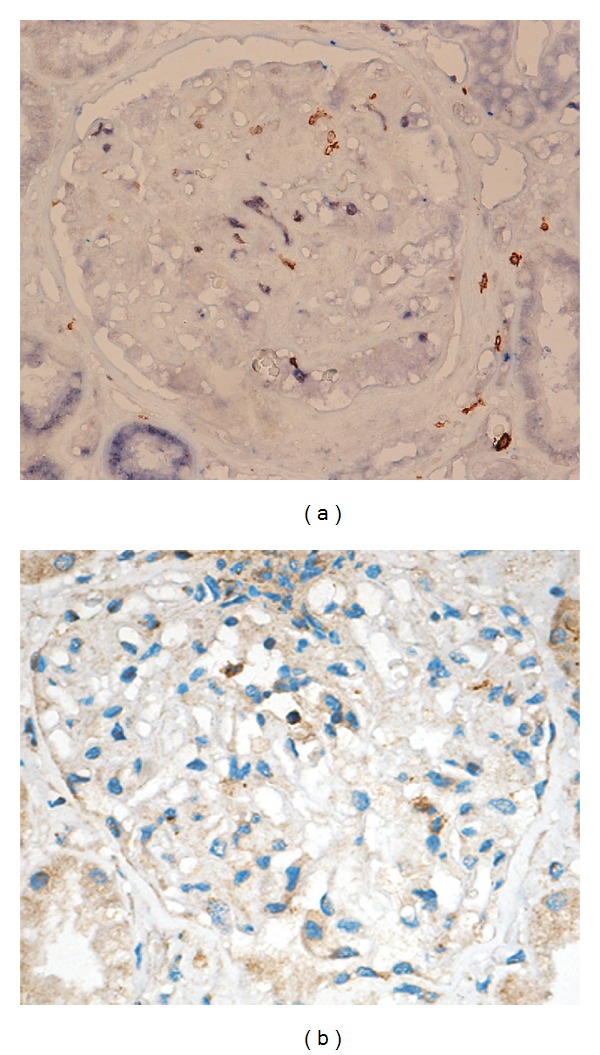
Immunohistological analysis of intraglomerular T-bet and GATA3. Positive staining is labeled in brown color. (a) Intraglomerular T-bet expression. (b) Intraglomerular GATA3 expression (magnification 400x).

The intragraft expression of T-bet and GATA3 was retrospectively studied via immunohistochemistry using stored residual biopsy tissues (Figure 1). Immunohistochemistry was performed on formalin-fixed, paraffin-embedded tissue. Regimens included mouse monoclonal antibody to T-bet (H-210, sc-21003; Santa Cruz Biotechnology, Santa Cruz, CA) rabbit polyclonal to GATA3 (ab61168; Abcam, Cambridge, UK) and mouse monoclonal antibodies to CD68 (KP1; Dako, Carpinteria, CA). Sections were reviewed by two separate pathologists, and the results were expressed as total number of positive cells per glomerulus or per square millimeter in the cortex.

### 2.4. Treatment of Acute Rejection

Once a rejection episode had occurred, bolus corticosteroid therapy (methylprednisolone 500 mg/day for 3 days) was selected as first-line treatment. Concomitantly, all the patients were given MMF (1.5 g/day) and Tac (trough levels maintained at 8–15 ng/mL). For patients being treated with Tac, MMF, and steroids as primary immunosuppression, the dose of Tac was increased so that trough levels were maintained at 8–15 ng/mL. If patients needed dialysis, continuous venovenous hemofiltration (CVVH) was performed. Immunoadsorption was used for patients with high level of antibodies or very strong and diffuse C4d staining. 

### 2.5. Statistical Methods

Descriptive statistical values are expressed as mean ± SD. Between-group differences in frequencies of clinical characteristics were determined using the Fisher exact test. The analyses were done using SPSS 15.0 (SPSS, Chicago, IL, USA). A *P* value of 0.05 or less was considered significant.

## 3. Results

### 3.1. General Information

Twenty-six renal allograft recipients who developed acute rejection were included in this study, including 11 cases of C4d-positive ABMR, 10 cases of T-cell-mediated acute rejection (TCMR), and 5 cases of C4d-negative ABMR. All the rejection episodes were proven by renal biopsy. The diagnoses of C4d-positive ABMR and TCMR were based on Banff, 2001 [6]. The diagnosis of C4d-negative ABMR was also based on Banff, 2001, except for C4d deposition. There were no retransplant cases in this cohort, and no patients were positive for panel-reactive antibodies (PRA) pretransplant. All the cases received IL-2R antibody as induction therapy and mycophenolate mofetil (MMF) + tacrolimus (Tac) + prednisolone as baseline immunosuppressants. There were no significant differences among groups in recipients' age, cold and warm ischemia time (Table 1).

### 3.2. T-bet and GATA3 Expressions Were Increased during Acute Rejection

We used immunohistochemistry to detect T-bet and GATA3 expressions; cells (excluding tubular epithelial cells) expressing T-bet and GATA3 were counted. In protocol biopsies from recipients with normal graft function (*n* = 6), neither T-bet nor GATA3 positive cells could be detected. However, in patients with acute rejection, both T-bet- and GATA3-expressing cells were significantly increased. All the patients had increased T-bet expression in interstitial area and 80.8% in glomerulus. GATA3 could be detected in 46.2% patients in interstitial area and 88.5% in glomerulus. Our observations were focused on the intraglomerular expression of T-bet and GATA3.

### 3.3. T-bet/GATA3>1 Distinguishes ABMR from TCMR

According to the ratio of intraglomerular T-bet/GATA3 positive cells, we divided the patients into two groups: T-bet/GATA3>1 and T-bet/GATA3≤1. There were no significant differences between patients' age, onset time of rejection, and induction and maintenance of immunosuppressants. However, we found that T-bet/GATA3>1 was strongly correlated with ABMR (93.3% versus 18.2%) and related lesions. The incidence of positive HLA-I/II antibodies (*P* < 0.001) and glomerulitis (*P* = 0.013) is significantly higher in T-bet/GATA3>1 group. The scores of PTC (*P* = 0.052) and glomerulitis (*P* < 0.001) were also higher in T-bet/GATA3>1 group (Table 2).

### 3.4. T-bet/GATA3>1 Is Strongly Correlated with C4d-Negative ABMR

We compared the characteristics of C4d-positive ABMR and C4d-negative ABMR (Table 3) and found that there were no differences between two groups in clinical and histological characteristics, such as incidences of HLA-I/II antibodies, incidence and severity of PTC inflammation as well as glomerulitis, and most importantly, the resistance of steroid treatment. When compared with TCMR group, C4d-negative ABMR group had significantly higher incidences of PTC inflammation and higher PTC score and glomerulitis score, which were very similar to C4d-positive ABMR. There was no difference in intraglomerular ratio of T-bet/GATA3 between the C4d-positive and -negative ABMR groups, while it was significantly higher in both groups compared with TCMR group. On the other hand, T-bet/GATA3 ratio >1 was 80% in the C4d-negative ABMR group, suggesting a diagnosis value of T-bet/GATA3 ratio in this special type of rejection.

### 3.5. T-bet/GATA3>1 Is Strongly Correlated with Steroid-Resistant Acute Rejection

 In T-bet/GATA3>1 group, only 1 rejection episode had positive response to steroid treatment, even for the patient in TCMR group, while, in the T-bet/GATA3≤1 group, 100% of the rejection episodes can be reversed by steroid treatment (*P* < 0.0001), including two cases of ABMR. Obviously, T-bet/GATA3>1 was strongly correlated with steroid-resistant acute rejection.

### 3.6. Sensitivity and Specificity

We compared the sensitivity and specificity of C4d and T-bet/GATA3 ratio as markers of ABMR; T-bet/GATA3>1 had a slight lower (90% versus 100%) specificity but a much higher (87.5% versus 68.8%) sensitivity compared with C4d.

## 4. Discussion

C4d deposition in PTC area has been regarded as a marker of ABMR for years [16, 17]. C4d-positive has been widely accepted as one of the diagnosis criteria [6, 18] and has contributed to the diagnosis and treatment of ABMR. However, as the recognition of a group of C4d-negative ABMR, C4d is no longer a reliable marker for ABMR diagnosis; thus, seeking a diagnosis marker that can distinguish C4d-negative ABMR is very important.

In our previous observation [15], we found that predominance of intraglomerular T-bet or GATA3 is correlated with different mechanisms of acute renal allograft rejection, suggesting that ratio of T-bet/GATA3 might be useful to distinguish ABMR from TCMR. T-bet expression is strongly correlated with peritubular capillaritis and glomerulitis, which are typical lesions of ABMR. The predominance of T-bet can also be found in transplant glomerulopathy, which is a chronic form of ABMR [19]. This study investigated the value of intraglomerular T-bet/GATA3 ratio in the diagnosis of ABMR. We found that the ratio of intraglomerular expression of T-bet/GATA3 can be used as a marker of ABMR.

Intraglomerular expression of T-bet/GATA3>1 is strongly correlated with ABMR. In acute rejection episodes with intraglomerular T-bet/GATA3>1, 93.3% of patients were ABMR, and only 1 patient (6.7%) was diagnosed as developing TCMR. Nonetheless, intraglomerular T-bet/GATA3>1 is strongly correlated with positive HLA-I/II antibodies and antibody-related lesions, such as glomerulitis, PTC score, and glomerulitis score. We need to point out that the correlation between T-bet/GATA3>1 and incidences of glomerulitis, PTC score, and glomerulitis score is even stronger than C4d deposition.

In order to evaluate the significance of T-bet/GATA3 in the diagnosis of C4d-negative ABMR, we compared the characteristics of C4d-positive ABMR, C4d-negative ABMR, and TCMR and found that there were no significant differences between C4d-positive and -negative ABMR in either incidence or severity of antibody-related lesions. The expression of T-bet and GATA3 and their ratio were very similar between the two groups. When compared with TCMR, there were significant differences between C4d-negative ABMR and TCMR, which is comparable to C4d-positive ABMR. These data proved that C4d-positive ABMR and C4d-negative ABMR shared the same characteristics.

As C4d-positive and -negative ABMR share similar T-bet and GATA3 expression, the ratio of T-bet/GATA3>1 is correlated with C4d-negative ABMR as well. The correlation of T-bet/GATA3>1 with C4d-negative ABMR resulted in a higher sensitivity in diagnosis of ABMR. In this group, compared with C4d deposition, T-bet/GATA3>1 had a similar specificity but a higher sensitivity in the diagnosis of overall ABMR. This suggests that T-bet/GATA3>1 is potentially a valuable maker for the diagnosis of ABMR, especially for the diagnosis of C4d-negative ABMR.

ABMR is featured to be resistant to steroid treatment [20]. Our data showed that intraglomerular ratio of T-bet/GATA3 is also correlated with response to the treatment of rejection. T-bet/GATA3>1 is associated with steroid resistance. It is interesting that, even in ABMR group, T-bet/GATA3<1 is correlated with a good response to steroid treatment. The resistance of steroid treatment in T-bet/GATA3>1 group is consistent with nature of ABMR.

The exact role of T-bet and GATA3 in the pathogenesis of ABMR remains unclear. It is possible that the high expression of T-bet expression in glomerulus will induce Th1 activity and resulted in intraglomerular macrophages infiltration, which is the feature of ABMR. As the sample size is rather small in this study, a prospective multiple centers study will be helpful to prove our findings in this preliminary study.

Overall, intraglomerular Th1/Th2 transcription factors T-bet/GATA3>1 are correlated with both C4d-positive and -negative ABMR. T-bet/GATA3>1 might be used as a diagnosis maker of ABMR in addition to C4d deposition. 

## Figures and Tables

**Table 1:** Clinical characteristics of patients participating in this study.

Characteristics	C4d(+) ABMR (*n* = 11)	C4d(−) ABMR (*n* = 5)	TCMR (*n* = 10)	*P* value
Gender, male (%)	2 (18.2)	4 (80.0)	9 (90.0)	0.002
Age (years)	40.18 ± 8.38	39.60 ± 12.95	38.80 ± 11.74	0.957
Donor age (years)	44.00 ± 9.89	44.90 ± 11.92	41.20 ± 10.84	0.771
Positive pretransplant PRA (*n*)	0	0	0	—
Previous transplant	0	0	0	—
Cold ischemic time (h)	8.18 ± 1.40	8.40 ± 1.14	8.93 ± 1.57	0.866
Warm ischemic time (min)	6.36 ± 1.20	6.20 ± 1.30	6.80 ± 1.31	0.623
Induction with IL-2R antibody, *n* (%)	11 (100)	5 (100)	10 (100)	—
Baseline immunosuppressants				0.118
MMF + Tac + Pred	11 (100)	5 (100)	10 (100)	—
Time of biopsy after Tx (day)	6.36 ± 5.16	3.40 ± 0.89	11.50 ± 4.90	0.008

PRA: panel-reactive antibody; IL: interleukin; MMF: mycophenolate mofetil; Pred: prednisolone; Tac: tacrolimus; Tx: transplantation; ABMR: antibody-mediated rejection; TCMR, T-cell-mediated rejection.

**Table 2:** Patients' demography and histologic characters with different T-bet/GATA3 ratios and status of C4d deposition.

	T-bet/GATA3>1 (*n* = 15)	T-bet/GATA3≤1 (*n* = 11)	*P*	C4d+ (*n* = 11)	C4d− (*n* = 15)	*P*
Gender (female %)	8 (53.3%)	3 (27.3%)	0.193	9 (81.8%)	2 (13.3)	<0.001
Age	39.40 ± 8.72	39.73 ± 12.48	0.938	40.18 ± 8.38	39.07 ± 11.69	0.790
Rejection type						
ABMR	14 (93.3%)	2 (18.2%)	<0.001	11 (100%)	5 (33.3%)	0.001
TCMR	1 (6.7%)	9 (81.8%)	<0.001	0	10 (66.7%)	0.001
Positive HLA-I or II Ab	14 (93.3%)	2 (18.2%)	<0.001	11 (100%)	5 (33.3%)	<0.001
Histological features						
Peritubular capillaritis	14 (93.3%)	7 (63.6%)	0.163	11 (100%)	10 (66.7%)	0.037
PTC score	1.93 ± 1.03	1.09 ± 1.04	0.052	2.00 ± 0.89	1.27 ± 1.16	0.094
Glomerulitis	15 (100%)	7 (63.6%)	0.013	11 (100%)	11 (73.3%)	0.068
Glomerulitis score	2.20 ± 0.94	0.73 ± 0.65	<0.001	2.18 ± 0.98	1.13 ± 0.99	0.013
Tubulitis	12 (80.0%)	11 (100%)	0.339	8 (72.7%)	14 (93.3%)	0.158
Tubulitis score	1.07 ± 0.80	1.64 ± 0.92	0.106	0.91 ± 0.70	1.60 ± 0.91	0.047
Intraglomerular CD68	9.03 ± 7.86	1.89 ± 4.09	0.006	9.49 ± 8.79	3.45 ± 5.01	0.059
Intraglomerular T-bet	2.66 ± 2.74	0.42 ± 0.48	0.007	2.67 ± 3.20	1.02 ± 1.18	0.077
Intraglomerular GATA3	0.69 ± 0.61	2.20 ± 1.41	0.001	0.73 ± 0.71	1.77 ± 1.41	0.034

ABMR: antibody-mediated rejection; TCMR, T-cell-mediated rejection.

**Table 3:** Histologic lesion of renal allograft.

	C4d(+) ABMR (*n* = 11)	C4d(−) ABMR (*n* = 5)	TCMR (*n* = 10)	*P*12	*P*13	*P*23
PTC inflammation, *n* (%)	11 (100%)	5 (100%)	4 (40%)	—	0.003	0.031
PTC score	2 ± 0.89	2.20 ± 1.10	0.80 ± 0.92	0.704	0.007	0.021
Glomerulitis, *n* (%)	11 (100%)	5 (100%)	6 (60%)	—	0.023	0.111
Glomerulitis score	2.18 ± 0.98	2.00 ± 1.00	0.70 ± 0.67	0.738	0.001	0.010
Tubulitis, *n* (%)	8 (72.7%)	4 (80%)	10 (100%)	0.763	0.082	0.157
Tubulitis score	0.91 ± 0.70	0.80 ± 0.45	2.00 ± 0.82	0.756	0.004	0.010
Intimal arteritis, *n* (%)	7 (63.6%)	3 (60%)	3 (30%)	0.893	0.133	0.280
Interstitial inflammation score	1.73 ± 0.65	2.4 ± 0.55	1.7 ± 0.67	0.064	0.926	0.067
Intraglomerular immunohistological analysis						
T-bet (cells/glomeruli)	2.67 ± 3.20	2.30 ± 1.19	0.37 ± 0.42	0.808	0.039	0.020
GATA3 (cells/glomeruli)	0.73 ± 0.71	1.32 ± 0.78	2.00 ± 1.63	0.156	0.042	0.401
T-bet/GATA3>1	9 (81.8%)	4 (80%)	1 (10%)	0.933	0.001	0.009
Interstitial immunohistological analysis						
T-bet (cells/mm^2^)	63.64 ± 65.88	77.6 ± 47.72	124.40 ± 112.66	0.679	0.158	0.280
GATA3 (cells/mm^2^)	20 ± 37.65	11.2 ± 20.86	27.2 ± 41.17	0.636	0.680	0.434
T-bet/GATA3>1	9 (81.8%)	5 (100%)	9 (90%)	0.324	0.602	0.480

ABMR: antibody-mediated rejection; TCMR, T-cell-mediated rejection.

*P*12 means *P* value for C4d(+) ABMR group and C4d(−) ABMR group, *P*13 means *P* value for C4d(+) ABMR group and TCMR group, *P*23 means *P* value for C4d(−) ABMR group and TCMR group.
